# Supplementary material for: GardeninA confers neuroprotection against environmental toxin in a Drosophila model of Parkinson’s disease
Source: Commun Biol. 2021 Feb 5;4:162. doi: 10.1038/s42003-021-01685-2 (PMC7864937; doi:10.1038/s42003-021-01685-2)
Supplement: Supplementary file 2 — Supplementary Information [file 42003_2021_1685_MOESM2_ESM.pdf]

Gardenin A confers neuroprotection against environmental toxin in a *Drosophila* model of Parkinson’s disease

Urmila Maitra[1*], Thomas Harding[1], Qiaoli Liang[2], Lukasz Ciesla[1*]

[1]Department of Biological Sciences, University of Alabama, 2320 Science and Engineering Complex, Tuscaloosa, Alabama 35487-0344, USA; [2]Mass Spectrometry Facility, Department of Chemistry and Biochemistry, University of Alabama, 2004 Shelby Hall, Tuscaloosa, Alabama 35487-0336, USA.

*Co-corresponding authors: Dr. Urmila Maitra and Dr. Lukasz Ciesla

Department of Biological Sciences,

University of Alabama,

2320 Science and Engineering Complex,

Tuscaloosa, Alabama 35487-0344, USA.

Email: lmciesla@ua.edu; umaitra@ua.edu

Tel: 205-348-1828

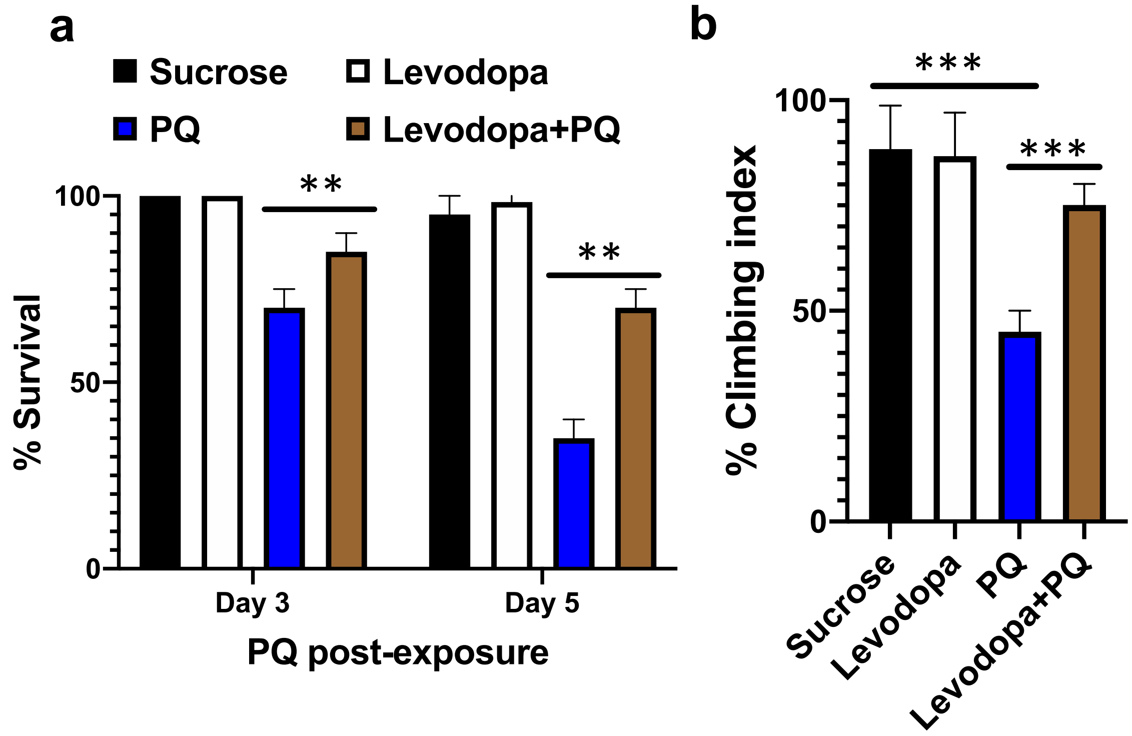

Figure S1. The protective effects of Levodopa against paraquat-induced toxicity and mobility defects in a *Drosophila* PD model.

a. Survival assays were set up using male flies following exposure to either sucrose or 5 mM PQ or 0.5 mM levodopa (L-dopa) or L-dopa+PQ and the number of live flies was recorded every 24 h and the survival percentages were plotted at the indicated time points (Days 3 and 5). Data are representative of ten independent biological replicates with 10 flies per feeding conditions. Data shown represent mean ± SEM. **$p<0.01$ based on the Mann-Whitney U test. b. Negative geotaxis assays were used to determine the protective effect of L-dopa on the climbing abilities of flies exposed to PQ. The number of flies able to cross 5 cm within 20 s were recorded and plotted at the specified time points (24 and 48 h). Data are representative of at least five independent experiments with 10 flies per group. ***$p < 0.001$ based on one-way ANOVA between indicated feeding conditions.

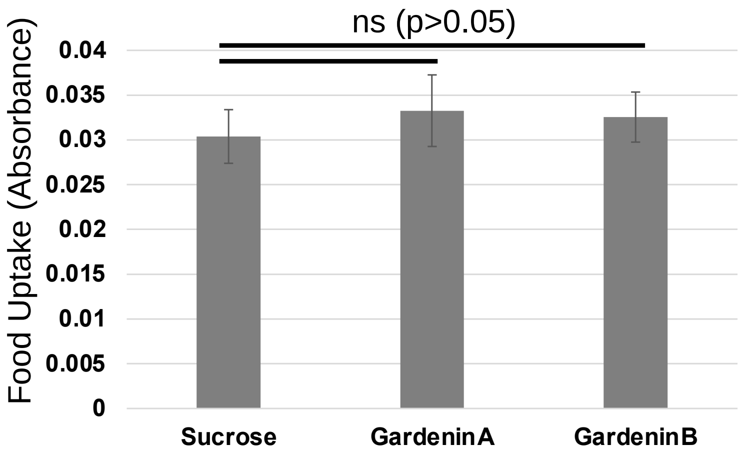

Figure S2. GardeninA or GardeninB pre-treatment does not affect food intake ability in *CantonS* wild-type flies.

The wild-type *CantonS* flies were fed either 2.5% sucrose or GardeninA or GardeninB containing the blue food dye (1% FD&C Blue#1) to quantitate food intake. The flies were washed and homogenized in 1X PBS containing 1% Triton X-100 followed by centrifugation at 12,000 rpm for 5 min and the supernatants were measured at OD630 nm. Data were analyzed using the two-tailed Student's *t-test* and error bars indicate the standard deviation. No statistically significant differences ($p>0.05$; ns=non-significant) in the food intake abilities were observed between the specified groups. Data are representative of five independent experiments with 10 male flies per treatment group.
